# Pulmonary versus Nonpulmonary Nontuberculous Mycobacteria, Ontario, Canada

**DOI:** 10.3201/eid2311.170959

**Published:** 2017-11

**Authors:** Sarah K. Brode, Alex Marchand-Austin, Frances B. Jamieson, Theodore K. Marras

**Affiliations:** West Park Healthcare Centre, Toronto, Ontario, Canada (S.K. Brode);; University of Toronto, Toronto (S.K. Brode, F.B. Jamieson, T.K. Marras);; University Health Network and Sinai Health System, Toronto (S.K. Brode, T.K. Marras);; Public Health Ontario, Toronto (A. Marchand-Austin, F.B. Jamieson)

**Keywords:** epidemiology, infection, nontuberculous mycobacterium, Mycobacterium avium complex, nontuberculous mycobacteria, tuberculosis and other mycobacteria, Ontario, Canada, NTM, bacteria

## Abstract

In Ontario, Canada, during 1998–2010, nontuberculous mycobacteria (NTM) from pulmonary sites comprised 96% of species/patient combinations isolated; annual rates of isolation and cases increased steadily. NTM isolates from nonpulmonary sites comprised 4% of species/patient combinations; annual rates and cases were temporally stable. NTM increases were driven exclusively by pulmonary isolates and disease.

Nontuberculous mycobacteria (NTM) cause pulmonary and nonpulmonary disease, but most isolates and disease cases are pulmonary (*1*). Studies have demonstrated temporal increases in pulmonary NTM isolation and disease (*2**,**3*). To determine trends in nonpulmonary NTM, we compared annual observed prevalence of pulmonary versus nonpulmonary NTM in Ontario, Canada, and compared the spectrum of NTM species isolated by body site.

## The Study

We retrospectively reviewed positive NTM culture results obtained during 1998–2010 by the Public Health Ontario Laboratory, which identifies >95% of NTM isolates in Ontario (*4*). Until mid-2000, cultures were performed by using a Bactec 460 TB system, after which a BACTEC MGIT 960 system (Becton Dickinson, Franklin Lakes, NJ, USA) was used. DNA probes (AccuProbe; Hologic Inc., Marlborough, MA, USA) were used for speciation of *Mycobacterium avium* complex (MAC) and *M. gordonae* isolates; during 1998–2007, high-performance liquid chromatography was used to speciate others; thereafter, DNA probes (AccuProbe and GenoType line-probe assays [Hain Lifescience GmbH, Nehren, Germany]) were used. Because MAC was not speciated before 2008, we used this designation throughout the study. 

We counted the persons for whom >1 positive culture for each NTM species/complex per year per body site was reported. Outcomes were pulmonary isolation (>1 positive culture from sputum, bronchoscopy samples, pleural fluid/tissue, or lung tissue); pulmonary disease (≥2 positive sputum cultures of the same species within the calendar year, or >1 positive culture from bronchoscopy, pleural fluid/tissue, or lung tissue (by American Thoracic Society microbiological definition, positive predictive value 70%–100%) (*5**–**8*); and nonpulmonary isolation (>1 positive culture from other sources). *M. gordonae* was considered a contaminant and excluded from pulmonary disease (*9*) but included in pulmonary isolation and nonpulmonary case counts. Outcomes were not mutually exclusive; we considered persons with NTM pulmonary disease to have pulmonary isolates and counted persons with pulmonary and nonpulmonary isolates in both groups. Careful electronic and manual selection ensured that each patient/species/anatomic site could be represented only once per year. We calculated prevalence of annual NTM isolation and NTM pulmonary disease as the number of persons from whom NTM was isolated or who had disease in a calendar year divided by the contemporary population (Statistics Canada, http://www.statcan.gc.ca/tables-tableaux/sum-som/l01/cst01/demo02a-eng.htm), expressed per 100,000 population. We used a generalized linear model with negative binomial distribution to assess annual rate changes and performed analyses with SAS version 9.4 (SAS Institute, Inc., Cary, NC, USA). All tests were 2-tailed with a type 1 error (α) rate of 5%. The University of Toronto Research Ethics Board approved this study.

During the study period, NTM was isolated from 26,067 patients. Pulmonary isolates predominated: mean annual unique species/patient/body site combinations were 2,631 pulmonary (96%) and 103 nonpulmonary (4%) (Table). Pulmonary and nonpulmonary NTM was isolated from 169 (0.6%) patients. Species distributions from pulmonary versus nonpulmonary sources were similar: MAC, followed by *M. xenopi*, from pulmonary and nonpulmonary sites, except for *M. marinum*, which was rarely pulmonary.

**Table:** Average number of patients per year who had nontuberculous mycobacteria isolated, by body site and species/complex, Ontario, Canada, 1998–2010*

Species	Pulm	Skin/soft tissue	MS	Lymph	Blood/marrow	GI/GU	CNS	Other
MAC	1,328	8	1	2	17	18	0.8	3
*Mycobacterium xenopi*	568	0.2	0.3	0	0.3	10	0.2	0.5
*M. gordonae*†	338	0.2	0	0	0	6	0.1	0.3
*M. fortuitum*	131	2	0.8	0	2	3	0.2	1.2
*M. abscessus*	58	2	0.4	0.1	0.5	0.3	0	0.8
*M. chelonae*	40	3	1	0.2	0.5	0.7	0	1.0
*M. simiae* complex	42	0.6	0.3	0.1	0.5	0.7	0	0.5
*M. kansasii*	34	0.3	0.2	0	0.2	0.6	0	0.1
*M. marinum*	0	6	1.5	0	0	0	0	0.6
Other‡	92	0.8	0.5	0.1	1	2	0.1	0.3
All	2,631	22	6	3	22	41	1	8

*Contemporary Ontario population 11.3–13.2 million. CNS, central nervous system; GI, gastrointestinal system; GU, genitourinary system; lymph, lymphatic system; MAC, *Mycobacterium avium* complex; MS, musculoskeletal system; pulm, pulmonary system. †*M. gordonae* was excluded from the number of pulmonary disease cases but included in pulmonary and nonpulmonary isolation cases. ‡Other species most commonly identified (% of grand total over entire study period, average no. patients per year), for pulmonary isolates were *M. mucogenicum* (0.93%, 24.5), *M. terrae* complex (0.43%, 11.4), *M. scrofulaceum* (19%, 5), *M. peregrinum* (0.17%, 4.4), *M. neoaurum* (0.14%, 3.8), *M. shimoidei* (0.13%, 3.3), *M. szulgai* (0.12%, 3.2), *M. celatum* (0.09%, 2.4), *M. malmoense* (0.08%, 2.2), *M. elephantis* (0.08%, 2.1), and *M. margeritense*/*M. smegmatis* (0.06% each, 1.5 each) and for nonpulmonary isolates included *M. mucogenicum* (1.3%, 1.38), *M. smegmatis* (1.0%, 1.08), *M. terrae* complex (0.5%, 0.54), *M. genavense* (0.4%, 0.38), *M. senegalense* (0.2%, 0.23), *M. malmoense*/*M. scrofulaceum*/*M. szulgai* (0.15% each, 0.15 each), *M. margeritense*/*M. shimoidei* /*M. elephantis* (0.07% each, 0.08 each).

**Figure 1:**
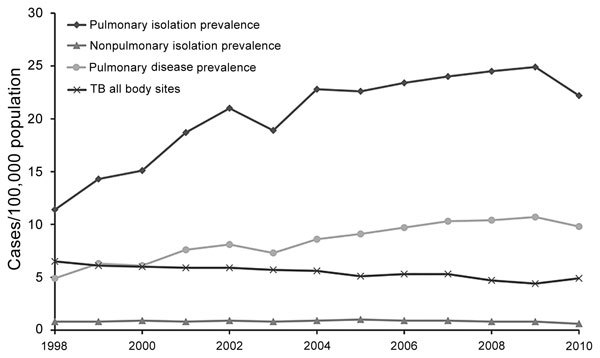
Prevalence of pulmonary and nonpulmonary nontuberculous mycobacteria (NTM) isolation and pulmonary NTM disease in Ontario, Canada, 1998–2010. Annual increase and modeled annual change were 6.3% (*3**,**4*) and 1.04 (95% CI 0.696–1.38)/100,000 population (p<0.001) for pulmonary isolation and 8.0% (*3*) and 0.402 (95% CI 0.307–0.497)/100,000 population (p<0.001) for pulmonary disease. Significant increases occurred in *Mycobacterium* *avium* complex (annual change 0.291 [95% CI 0.236–0.346]/100,000 population; p<0.001); *M. xenopi* (annual change 0.059 [95% CI 0.015–0.103]/100,000 population; p = 0.002); and *M. abscessus* (annual change 0.019 [95% CI 0.015–0.024]/100,000 population; p<0.001). TB (all body sites) isolation decreased by an average of 2.2% annually (6.5 to 4.9/100,000 population) during the study period. TB, tuberculosis.

**Figure 2:**
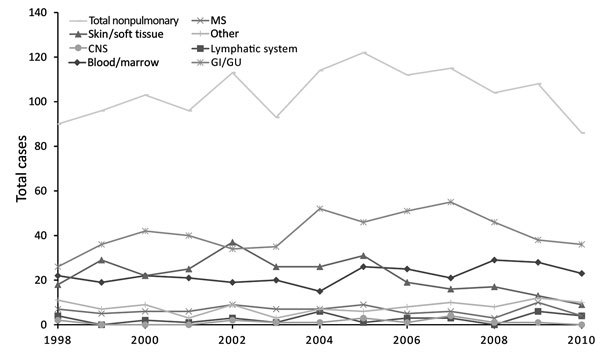
Isolation of nonpulmonary nontuberculous mycobacteria by body site, Ontario, Canada, 1998–2010. There was no significant temporal change by anatomic site except for a decrease in skin/soft tissue infections (modeled annual change −0.011 [95% CI −0.020 to −0.003]/100,000 population; p = 0.001). *Mycobacterium marinum* significantly decreased over time (modeled annual change −0.003 [95% CI −0.007 to 0.001]/100,000 population; p = 0.0480); isolation of other species from nonpulmonary sites was unchanged. Overall nonpulmonary isolation modeled annual change was −0.004 (95% CI −0.019 to 0.010)/100,000 population (p = 0.410). CNS, central nervous system; GI, gastrointestinal system; GU, genitourinary system, lymph, lymphatic system; MS, musculoskeletal system.

Annual rates of pulmonary NTM isolation and disease prevalence were 11.4 isolates and 4.65 cases per 100,000 population in 1998 and 22.2 and 9.08 per 100,000 in 2010 (Figure 1). As reported, frequency of pulmonary isolation and disease increased steadily (*3**,**4*). Annual prevalence of nonpulmonary NTM was 0.65–0.79 isolates/100,000 and did not change appreciably over time (Figure 2).

## Conclusions

Contrasting with documented increased pulmonary NTM isolation and disease in Ontario, rates of nonpulmonary NTM isolation are stable or decreasing, regardless of species or body site. The difference in trends indicates that pulmonary and nonpulmonary NTM represent different diseases with different risk factors. NTM pulmonary disease generally occurs in persons with preexisting structural lung damage (*8*) or abnormal mucociliary function (*10*) and is strongly associated with increasing age (*5*). Nonpulmonary NTM disease occurs in persons with generalized immunosuppression or after NTM entry into breached tissue (*9*). We considered the possibility that after the introduction of antiretroviral medication for HIV in the 1990s, a reduction in disseminated NTM may have masked an increase in nonpulmonary NTM infection in non–HIV-infected patients. However, the absence of decreased isolation from blood or bone marrow in the first several years and the steady number of cases (Figure 2) do not support this possibility. Although we lacked data regarding immune status, the small proportion of patients from whom pulmonary and nonpulmonary NTM were isolated (0.6%) does not suggest a large proportion of severely immunosuppressed patients.

There are several possible explanations for discordant temporal trends between pulmonary and nonpulmonary NTM. A selective increase in respiratory exposure would preferentially drive pulmonary NTM. However, although exposure to respirable waterborne NTM is undoubtedly widespread, data identifying recent increases are lacking. Other possibilities include changes in risk factors for NTM pulmonary disease (e.g., aging, chronic obstructive pulmonary disease, and iatrogenic causes [medications]) and improved diagnostic modalities (e.g., increased use of computed tomography in the United States [*11*]). Reduced turberculosis incidence, leading to reduced cross-immunity to NTM, has been proposed as an explanation for the reciprocal trends in tuberculosis and NTM infections observed in many areas (*2*); however, it is not known why waning immunity would result in increased pulmonary NTM only.

A previous population-based study compared the epidemiology of pulmonary and nonpulmonary NTM by examining isolates referred to the Netherlands national reference laboratory during 2000–2007 (*12*). The study indicated large increased numbers of NTM isolates, mostly *M. avium*. The average annual percentage increase was similar for pulmonary (31.3%) and extrapulmonary (33.0%) *M. avium*, differing markedly from our results. The differences could reflect referral bias (the Netherlands national reference laboratory received 85% of NTM isolates; the Public Health Ontario Laboratory received >95%), improvements in laboratory methods in the Netherlands potentially increasing detection of both pulmonary and nonpulmonary NTM, or differences in NTM epidemiology by region (*13**,**14*).

In Olmsted County, Minnesota, USA, incidence of cutaneous NTM infections apparently tripled from 1980–1999 to 2000–2009, driven partly by increased *M. abscessus/chelonae*, often from surgical/cosmetic procedures (*15*). Contrasting with our observation of reduced skin/soft tissue infections, their observations could result from differences in methods, geography, or number of surgical/cosmetic procedures.

A study limitation is lack of clinical data to confirm NTM pulmonary disease. Although our definition of NTM pulmonary disease has acceptable positive predictive value (*5**–**8*), it misclassifies some patients as having disease. By contrast, we underestimated NTM pulmonary disease because we counted only persons who met the definition each calendar year; there were probably persons with prevalent disease that was undiagnosed or diagnosed in a previous year but lacked ongoing sputum collection or for whom NTM isolation was staggered over 2 calendar years. The net effect of over/underestimating NTM pulmonary disease is unclear. We also classified all nonpulmonary isolates as representing disease, overestimating nonpulmonary disease, especially because feces and urine comprise most gastrointestinal/genitourinary sources. Excluding gastrointestinal/genitourinary isolates would increase the proportion of pulmonary isolations from 96.0% to 97.7%. Although gastrointestinal/genitourinary isolates comprise the largest nonpulmonary group, the trend for this group is similar to that for others (Figure 2), so its inclusion does not affect our conclusions. The lack of data regarding the number of samples submitted prevents assessment of the effect of sampling on NTM isolation. However, we previously identified an increased number of pulmonary samples submitted during 1997–2002, leveling off during 2002–2007 (*4*). Given the steady increase in pulmonary isolates and disease throughout the study period, increased sampling does not explain our findings. Our lack of clinical data prevents comparison of features of pulmonary versus nonpulmonary NTM, and our lack of detailed epidemiologic data prevents assessment for regional or temporal nonpulmonary outbreaks from common point sources. The increase of NTM in Ontario reflects only pulmonary NTM.
